# The Ringleaders: Understanding the Apicomplexan Basal Complex Through Comparison to Established Contractile Ring Systems

**DOI:** 10.3389/fcimb.2021.656976

**Published:** 2021-04-19

**Authors:** Alexander A. Morano, Jeffrey D. Dvorin

**Affiliations:** ^1^ Biological and Biomedical Sciences, Harvard Medical School, Boston, MA, United States; ^2^ Division of Infectious Diseases, Boston Children’s Hospital, Boston, MA, United States; ^3^ Department of Pediatrics, Harvard Medical School, Boston, MA, United States

**Keywords:** cytokinesis, contractile ring, basal complex, Apicomplexans, actomyosin, division, schizogony, endodyogeny

## Abstract

The actomyosin contractile ring is a key feature of eukaryotic cytokinesis, conserved across many eukaryotic kingdoms. Recent research into the cell biology of the divergent eukaryotic clade Apicomplexa has revealed a contractile ring structure required for asexual division in the medically relevant genera *Toxoplasma* and *Plasmodium*; however, the structure of the contractile ring, known as the basal complex in these parasites, remains poorly characterized and in the absence of a myosin II homolog, it is unclear how the force required of a cytokinetic contractile ring is generated. Here, we review the literature on the basal complex in Apicomplexans, summarizing what is known about its formation and function, and attempt to provide possible answers to this question and suggest new avenues of study by comparing the Apicomplexan basal complex to well-studied, established cytokinetic contractile rings and their mechanisms in organisms such as *S. cerevisiae* and *D. melanogaster*. We also compare the basal complex to structures formed during mitochondrial and plastid division and cytokinetic mechanisms of organisms beyond the Opisthokonts, considering Apicomplexan diversity and divergence.

## Introduction

The Apicomplexa are a phylum of obligate intracellular parasites, categorized within the SAR (Stramenopile, Alveolata, Rhizaria) eukaryotes (Gräf et al., 2015). *Toxoplasma* and *Plasmodium*, two genera within the Apicomplexan phylum, are responsible for a significant degree of humanity’s infectious disease burden. While much progress has been made in the control of malaria over the past fifteen years, it still takes over 400,000 lives a year, and toxoplasmosis remains one of the world’s most widespread parasitic infections, posing significant danger to the pregnant and/or immunocompromised (Furtado et al., 2011; World Malaria Report 2019). Apicomplexans are also so divergent from the organisms in which most cell biology studies are done such as *Saccharomyces cerevisiae, Drosophila rerio*, *Caenorhabditis elegans*, etc., that basic cellular processes remain poorly understood. Cell division, for example, is incredibly complex in these organisms since their life cycles involve multiple morphologically diverse stages with both sexual and asexual replication. While most eukaryotes undergo simple cytokinesis where one cell produces two daughter cells, these pathogenic Some Apicomplexans utilize a divergent process known as schizogony wherein several successive rounds of karyokinesis result in a multinucleated schizont. The final karyokinesis is paired with cytokinesis, as the cytoplasmic mass segments into many daughter cells, each containing one nucleus. We note that *Toxoplasma* divides by schizogony in its definitive host in the process of producing gametes, but divides by endodyogeny, a single closed mitosis process where the daughter cells are assembled internally, during asexual replication; *Plasmodium* divide *via* schizogony during the asexual intra-erythrocytic stage of its life cycle (Francia and Striepen, 2014).

The molecular mechanisms underlying schizogony and how they relate to higher eukaryote division processes remain unknown. Possible parallels to the actomyosin ring-mediated cell division that characterizes most eukaryotic cytokinesis are difficult to identify as a result of Apicomplexan divergence and unique life cycle and division processes. Among the most significant differences at the genetic level is that Apicomplexans lack a homolog to the conventional myosin involved in actomyosin-mediated cell division, myosin II, and must utilize a different, yet uncharacterized, mechanism (Frénal et al., 2020). Recent research has begun to shed light on Apicomplexan cellular division, however, and revealed an important molecular machine, the basal complex, which likely acts as a contractile ring during asexual division.

Considering the phylogenetic distance between Apicomplexans and well-studied organisms such as yeast, it is likely not possible to find direct homologs in the latter for basal complex proteins, or vice versa. However, examining contractile ring composition in these organisms in comparison to the Apicomplexan basal complex may illuminate new avenues of study. While apicomplexans lack myosin II, for example, the ‘non-canonical’ myosins in *Plasmodium* may take on a myosin II-like role in the basal complex. Identifying similarities between other contractile rings and the basal complex may also identify Apicomplexan proteins important for basal complex function.

### The Minimal Contractile Ring

Before discussing the basal complex and how it compares to other eukaryotes’ contractile rings, it is useful to understand what a well-studied contractile ring usually consists of and how it functions in cellular division. In eukaryotes, the contractile ring is a structure that functions to generate constricting force required to separate daughter cells during cell division. It is assembled during cytokinesis and though it contains different accessory or regulatory proteins required for its assembly and function, the core proteins of the contractile ring are, in the vast majority of eukaryotes in which the contractile ring has been extensively studied, actin filaments (F-actin) and the myosin II motor protein. Its reliance on actin filaments means that actin filament stabilizing and destabilizing proteins are often important to proper formation and function, and because the contractile ring must assemble and constrict during a very specific late stage in the cell cycle, the processes underlying its assembly and function are often tightly temporally controlled by variations in mitotic kinase levels and phosphorylation/dephosphorylation signals (Miller, 2011).

The contractile ring as it exists in most eukaryotes requires both structural proteins (actin) and proteins that can generate force on actin fibers (myosin II), but even the most minimal contractile ring systems require at least a few additional components (Mangione and Gould, 2019). Detailing the minimal requirements, to the best of our current knowledge, for a contractile ring’s formation and function will provide a baseline against which *in vivo* contractile rings in both well-studied organisms and the more enigmatic Apicomplexan structures can be compared.

*In vitro* contractile rings have not yet been reconstituted from recombinant proteins alone, but functional structures approximate to a ‘minimal contractile ring’ have been purified from yeast spheroplasts. Rings isolated from *S. cerevisiae* cells *via* differential centrifugation and fractionation contained, in addition to actin and myosin, IQGAP (Iqg1p), actin, mlc2p (myosin regulatory light chain), cytokinetic F-BAR protein Hof1p, and the septin filament marker Cdc11p (Young et al., 2010). The rings retained some constrictive ability; thus this can be considered the ‘minimal’ apparatus required for *S. cerevisiae* ring formation and function (Young et al., 2010) (**Table 1**).

**Table 1:** To provide a general overview of which components of different eukaryotes’ contractile rings are conserved in Apicomplexans, we compiled a list of proteins important for the formation and function of each eukaryote’s contractile ring.

Contractile Ring Component	Stage of Contraction	*Plasmodium homolog?*	*Toxoplasma homolog?*
**Minimal contractile ring (*S. cerevisiae*)**
ACT1 (Actin)	Contraction	Actin I (PF3D7_1246200)	Actin ACT1 (TGME49_209030)
MYO2 (Myosin II heavy chain)	Contraction	NA*	NA*
IQG1P (IQGAP)	Assembly	NA	NA
MLC2 (myosin light chain 2)	Assembly	NA*	NA*
HOF1 (formin-binding)	Assembly	NA	NA
CDC3, CDC10, CDC11, CDC12, SH3 (Septins)	Assembly	NA	NA
CDC8 (Tropomyosin)	Contraction	NA	NA
***S. pombe* contractile ring**
mid1	Assembly	NA	NA
arp2/3	Assembly	NA*	NA*
for3 (formin)	Assembly	Formin 1 (PF3D7_0530900)	Formin FRM1 (TGME49_206430)
cdc12 (formin)	Assembly	Formin 2 (PF3D7_1219000)	Formin FRM2 (TGME49_206580)
fim1 (fimbrin)	Assembly	NA	NA
cof1 (cofilin)	Assembly	ADF1 (PF3D7_0503400), ADF2 (PF3D7_1361400)	ADF (TGME49_220400)
cdr2	Assembly	NA*	NA*
rga7	Contraction	NA	NA
cdc15 (F-Bar)	Assembly	NA	NA
rng10	Contraction	NA	NA
cdc3 (profilin)	Contraction	Profilin (PF3D7_0932200**)	Profilin PRF (TGME49_293690)
wsp1	Assembly	NA*	NA*
ppb1	Assembly	PF3D7_0802800	TGME49_311310
mid2 (anillin)	Assembly	NA	NA
rho1	Assembly	NA*	NA*
ain1 (actin crosslinker a-actinin)	Assembly/Contraction	NA	NA
pxl1 (paxillin homolog)	Assembly	NA	NA
plo1	Assembly	NA*	NA*
cdc7	Assembly	NA*	NA*
rgf3 (RhoGEF)	Assembly	NA	NA
cdc42 (Rho)	Assembly	NA*	NA*
art1 (RhoGEF)	Assembly	NA	NA
**Metazoan cytokinetic contractile ring**
ANLN (Anillin)	Contraction	NA	NA
RHOA (Rho GTPase)	Assembly	NA*	NA*
KIF23 ((m)KLP-1)	Assembly	Kinesin-8, putative (PF3D7_0111000)^§^	Kinesin motor-domain containing protein (TGME49_249020), (TGME49_319710), Kinesin-like protein (TGME49_211910), (286660)
RACGAP1/MgcRacGAP/CYK4	Assembly	NA	NA
ECT2	Assembly	NA*	NA*
ARHGEF2/GEF-H1	Assembly	NA	NA
PLEKHG6/MyoGEF	Assembly	NA	NA
ARHGAP1; p50 Rho-GAP	Assembly	NA	NA
RAC1	Assembly	NA	NA
CDC42 (CDC42 homolog)	Assembly	NA*	NA*
CDK1 (Cyclin B/Cdc2)	Assembly	Protein kinase 5 (PF3D7_1356900), Cdc2-related protein kinase 1 (PF3D7_0417800)	Protein Kinase domain -containing protein (TGME49_218220)
PLK1 (Polo-like kinase 1)	Assembly	NA*	NA*
AURKB (Aurora B kinase)	Assembly	PfArk1 (PF3D7_0605300), PfArk2 (PF3D7_0309200), PfArk3 (PF3D7_1356800)**	TgArk1 (TGME49_210280), TgArk2 (TGME49_318770), TgArk3 (TGME49_203010)**
DIAPH3 (protein diaphanous homolog 3, mDia2 (formin))	Assembly	Formin 1 (PF3D7_0530900), Formin 2 (PF3D7_1219000)	Formin FRM1 (TGME49_206430), Formin FRM2 (TGME49_206580)
ROCK1 (Rho-associated protein kinase 1)	Assembly	NA*	NA*
CIT (Citron Rho-interacting kinase)	Assembly	NA*	NA*
MYL12A (Myosin regulatory light chain 12A; myosin regulatory light chain 2, myosin rlc)	Contraction	NA*	NA*
PFN1 (Profilin 1)	Contraction	PF3D7_0932200**	Profilin PRF (TGME49_293690)**
CFL1 (Cofilin-1)	Contraction	ADF1 (PF3D7_0503400), ADF2 (PF3D7_1361400)	ADF (TGME49_220400)
ACTR2/3 (Arp2/3; Actin related protein 2/3)	Assembly	NA*	NA*
MYL1 (Myosin II essential light chain)	Contraction	NA*	NA*
**Mitochondrial fission contractile ring protein**
ACTR2/3 (Arp2/3; Actin related protein 2/3)	Assembly	NA*	NA*
CFL1 (Cofilin-1)	Contraction	ADF1 (PF3D7_0503400), ADF2 (PF3D7_1361400)	ADF (TGME49_220400)
CTTN (Src substrate cortactin)	Assembly	NA	NA*
DNM1L (Dynamin-1-like protein; Drp1)	Assembly	DrpC (PF3D7_1218500)	DrpC (TGME49_270690)
FIS1 (mitochondrial fission 1 protein)	Contraction	Fis1 (PF3D7_1325600)	Fis1 (TGME49_2633230)
MFF (mitochondrial fission factor)	Assembly	NA	NA
MIEF2 (Mitochondrial dynamics protein Mid49)	Assembly	NA	NA
MIEF1(Mitochondrial dynamics protein Mid51)	Assembly	NA	NA
INF2 (Inverted formin-2)	Assembly	Formin 1 (PF3D7_0530900), Formin 2 (PF3D7_1219000)	Formin FRM1 (TGME49_206430), Formin FRM2 (TGME49_206580)
CAF4 (CCr4-associated factor 4)	Assembly	NA*	NA*
DNM2 (Dynamin-2)	Contraction	DYN1 (PF3D7_1145400), DYN2 (PF3D7_1037500)	DrpA (TGME49_267800), DrpB (TGME49_321620)
**Plastid Division (in photosynthetic eukaryotes)**
FTSZ1(A) (Cell division protein FtsZ homolog 1, chloroplastic)	Contraction	Tubulin beta chain (PF3D7_1008700)**	Tubulin alpha chain (TGME49_231770), (TGME49_316400), (TGME49_231400)
FTSZ2(B) ((Cell division protein FtsZ homolog 2, chloroplastic)	Contraction	Tubulin beta chain (PF3D7_1008700)**	Tubulin beta chain (TGME49_221620), (TGME49_212240), (TGME49_266960)
ARC5 (Dynamin-like protein ARC5)	Contraction	DYN1 (PF3D7_1145400), DYN2 (PF3D7_1037500)	DrpA (TGME49_267800), DrpB (TGME49_321620), DrpC (TGME49_270690)
PDR1 (plastid division protein PDR1)	Assembly	NA	NA
DNM1 (dynamin-related GTPase)	Contraction	DYN1 (PF3D7_1145400), DYN2 (PF3D7_1037500)	DrpA (TGME49_267800), DrpB (TGME49_321620), DrpC (TGME49_270690)
MCD1 (multiple chloroplast division site 1)	Assembly	NA	NA
MIND1 (septum site-determining protein minD homolog, chloroplastic)	Assembly	NA	NA
MINE1 (cell division topological specficity factor homolog, chloroplastic)	Assembly	NA	NA
CDP1 (Plastid division protein CDP1, chloroplastic (Arc6))	Assembly	PF3D7_0803200^(¶)^	NA
PDV1 (plastid division protein PDV1)	Assembly	NA	NA
PDV2 (plastid division protein PDV2)	Assembly	NA	NA
**Dictyostelium division/wound closure proteins**
dymA (dynamin-A)	Contraction	DYN1 (PF3D7_1145400), DYN2 (PF3D7_1037500)	DrpA (TGME49_267800), DrpB (TGME49_321620)
dlpA (dynamin-like protein A)	Assembly	DrpC (PF3D7_1218500)	DrpC (TGME49_270690)
dlpB (dynamin-like protein B)	Assembly	DrpC (PF3D7_1218500)	DrpC (TGME49_270690)
calA (calmodulin)	Assembly	Calmodulin (PF3D7_1434200)	Calmodulin CAM1 (TGME49_246930), Calmodulin CAM2 (TGME49_262010)
nxnA (Annexin A7)	Contraction	NA	NA

*proteins identified through BLAST but identified as homologs of other proteins in the literature previously.

**not a homolog identified through BLAST but listed protein found as a homolog via literature search.

^§^maybe a homolog; low e-value and no other characterization but no definitive assignment yet.

^¶^identified through an OrthoDB search instead of NCBI BLAST.

We searched for homologous proteins in both *P. falciparum* and *T. gondii* using the BLAST tool on each organism’s database with the parameters described (criteria for identification of putative homolog was E value < 0.001). We bolstered the results of our simple search by examining the literature as well, taking into consideration homologs that had been identified *via* more stringent or comprehensive methods, allowing us to assign additional proteins as Apicomplexan homologs that did not meet our BLAST criteria and to discard other Apicomplexan proteins that fit the criteria because they possessed a certain conserved domain common to a class of proteins but had, through stricter analysis, previously been identified as homologs of other proteins within that class (e.g. protein kinases or phosphatases).

In less stringent *in vitro* conditions, the contractile rings of permeabilized *Schizosaccharomyces pombe* spheroplasts contained myosin II, actin, and tropomyosin Cdc8p (Mishra et al., 2013; Mabuchi et al., 2017). These rings too could constrict in the absence of other cytoplasmic components, provided ATP hydrolysis was uninhibited. Though mutants deficient in multiple proteins involved in the formation and function of the cytokinetic ring were made, only inhibition of ATP hydrolysis, tropomyosin, and actin crosslinking activity rendered the rings unable to form or constrict (Mishra et al., 2013). These ‘minimal’ contractile rings, therefore, required only F-actin, myosin II, ATPase activity, and actin crosslinking (**Table 1**).

This minimal contractile ring apparatus can generate force in the absence of accessory proteins. In one model, myosin mini-filaments are formed when the head region binds to actin and two heavy chains dimerize and interact with essential light chains and regulatory chains to form a functional hexamer unit, which can further polymerize (Schwayer et al., 2016; Pollard and O’Shaughnessy, 2019). Actin filaments are bound by these myosin ‘mini-filaments’ which pull anti-parallel actin strands together and reduce the ring diameter. A second model suggests depolymerization of actin filaments cross-linked by proteins bound to their minus ends could generate tension and drive ring constriction (Schwayer et al., 2016). In this model, myosin pulls on filaments adjacent to those depolymerizing, triggering disassembly of the cross-linked actin filaments (Zumdieck et al., 2007; Pinto et al., 2013; Lenz, 2020).

These hypothesized force generation mechanisms are unlikely to have exact parallels in Apicomplexans, considering how divergent Apicomplexans are from other eukaryotes. *Plasmodium falciparum*’s actin protein, for example, is only 70-80% similar to *S. cerevisiae* actin whereas human actin is over 90% similar (Das et al., 2017). Since Apicomplexans lack myosin II, it is difficult to speculate on the contribution of their non-canonical myosins to this process. Even if Apicomplexan myosins do not generate force during segmentation, actin depolymerization alone could potentially generate enough force to induce constriction, utilizing similar protrusive force as required for swift movement of *Listeria* (Mogilner et al., 2003; Ennomani et al., 2016). However, even this “elastic Brownian ratchet” mechanism requires the presence of ActA, Arp2/3, ADF, cofilin, and members of the Ena/Mena/VASP family, some of which have no *Plasmodium* homologues (**Table 1**). As previously mentioned, disrupting actin networks during division resulted in mis-localization of basal complex proteins in Toxoplasma, bolstering the idea that actin networks and actin depolymerization may play a significant role in the formation/contraction of the ring. Ultimately, more basal complex proteins need to be identified before a mechanism can confidently be proposed.

## The Basal Complex: Apicomplexans’ Cytokinetic Contractile Ring

Apicomplexa possess a structure analogous to the actomyosin ring common to eukaryotic cytokinesis, the basal complex, but it is still only hypothesized to act as a contractile ring. The basal complex is novel but poorly understood. While the structural details of cell division in Apicomplexa have been thoroughly examined using transmission electron microscopy, only recently have advances in genetics and microscopy enabled the molecular and mechanistic basis of these processes to begin to be understood in these organisms (Trager et al., 1966; Sheffield and Melton, 1968; Aikawa, 1971; Striepen et al., 2007). It is clear though that the proper formation and function of the basal complex is essential for successful segmentation in *Toxoplasma* and *Plasmodium* (Hu, 2008; Rudlaff et al., 2019).

**Figure 1:**
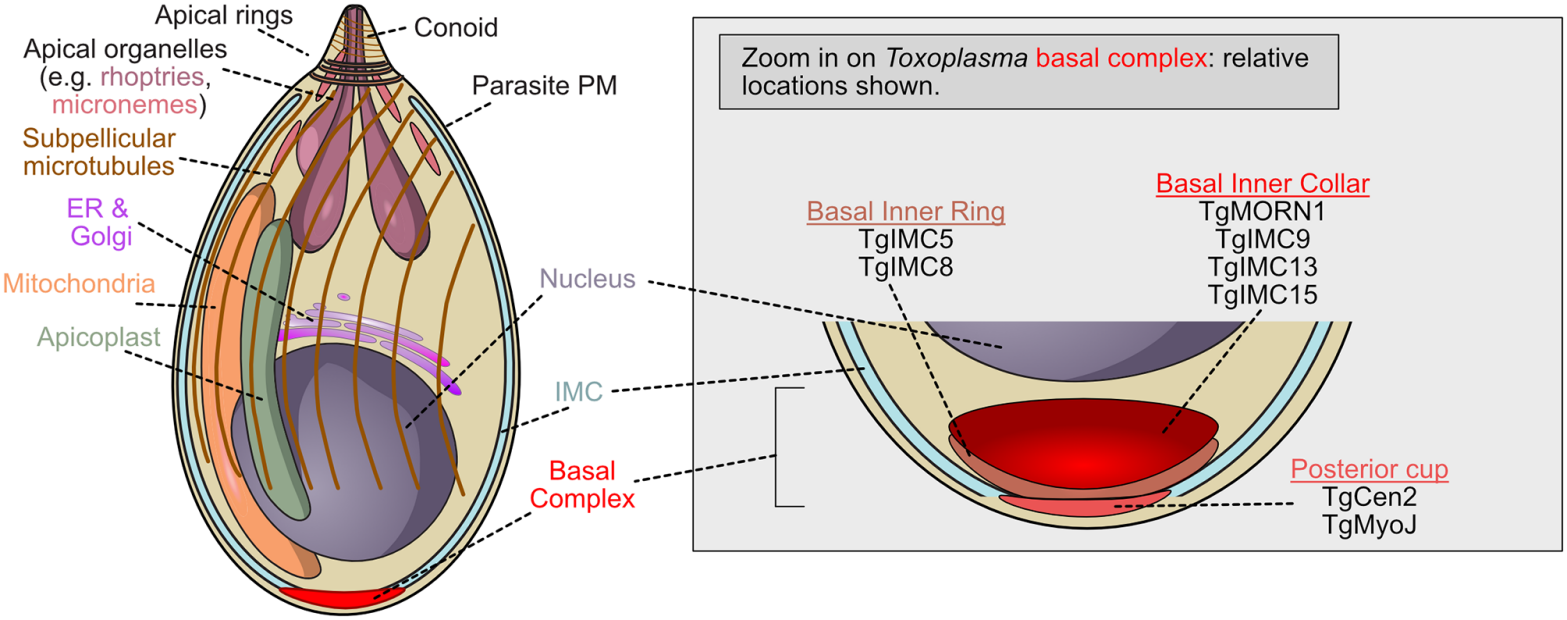
Schematic of *T. gondii* tachyzoite demonstrating the localization of basal complex proteins. *Toxoplasma gondii* is the Apicomplexan whose cell biology is best understood, but molecular function of the basal complex components remain poorly understood. Many basal complex proteins have been identified but little is known about their organization or function. The zoomed view of the basal complex is shown when cytokinesis is complete (or nearly complete). At this stage, TgMORN1, one of the first proteins recruited to the complex, is located with TgIMC9, TgIMC13, and TgIMC15 in the basal inner collar. The posterior cup contains two proteins which may provide contractile force, TgMyoJ and TgCentrin2. Between these two groups of proteins, TgIMC5 and TgIMC8 localize to the basal inner ring.

**Figure 2:**
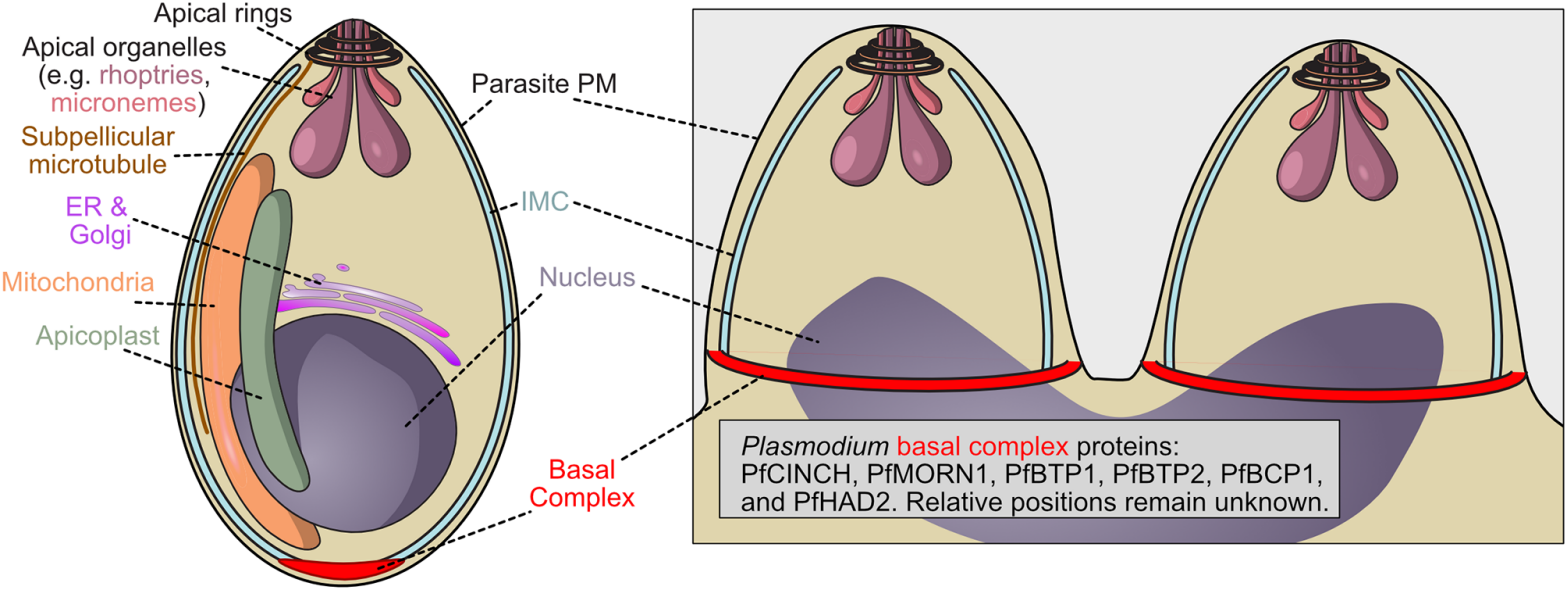
Schematic of *P. falciparum* merozoite demonstrating the localization of the basal complex. The basal complex of *Plasmodium falciparum* has not been as extensively characterized as that of *Toxoplasma*. Proteins PfMORN1, PfBTP1, PfCINCH, PfBTP2, PfBCP1, and PfHAD2 have been identified as members of the basal complex, visualized at the leading edge of the inner membrane complex, but their function, relative location within the structure, and order of assembly remain unknown.

The description of the basal complex in Apicomplexan parasites requires some basic definitions and descriptions of cellular structures specific to Apicomplexa and required for cytokinesis. (**Figures 1**, **2**). The Apicomplexan cortical cytoskeleton includes a unique, specialized membrane system immediately beneath the plasma membrane and composed of flattened vesicles or alveoli, known as the inner membrane complex. The inner membrane complex, or IMC, is made of multiple fused membrane ‘plates’ in *Toxoplasma* spp but in *Plasmodium* (aside from the transmission gametocyte stage), the IMC is made of a single vesicle (Porchet and Torpier, 1977; Bannister and Mitchell, 1995; Kono et al., 2012; Harding and Meissner, 2014). This membrane system provides structure, shape, and stability to the parasite, acts as a scaffold for proteins both within and attached to the membrane system during division, and is where the force generating complex required for invasion is located (Bergman et al., 2003; Baum et al., 2006). Beneath the flattened vesicles of the IMC, the subpellicular network (SPN) made of interwoven filaments of proteins known as alveolins, which are similar to intermediate filaments, stabilizes this system (Mann and Beckers, 2001). They also help maintain the shape and structure of the parasite and may be involved in other processes. Internal to the SPN, the Apicomplexans have one or more subpellicular microtubules that extend longitudinally down the long axis of the tachyzoite in *Toxoplasma* or the merozoite in *Plasmodium*.

### The *Toxoplasma gondii* Basal Complex

*Toxoplasma* parasites replicate asexually in a process called ‘endodyogeny’, wherein two daughter cells are formed inside the parent (Goldman et al., 1958; Sheffield and Melton, 1968; van den Zypen and Piekarski, 1968). In the intestinal epithelium of the cat, the definitive host, *Toxoplasma* reproduces *via* schizogony, like *Plasmodium*, and the released merozoites begin gamete formation (Aikawa, 1971; Dubey et al., 1998). However, these stages of Toxoplasma development have been studied much less than the asexual replication of Toxoplasma tachyzoites in intermediate hosts, and much less is known about this process, so this overview will focus on that stage.

During endodyogeny, centrosome replication occurs in the late G1 and early S phase, and the replicated centrosome helps assemble microtubules, the spindle, and the daughter cytoskeleton in general (Francia et al., 2012; Morlon-Guyot et al., 2016). Immediately following centrosome replication, the mitotic spindle begins to assemble and cytokinesis begins. The daughter basal complex forms relatively early in endodyogeny, before the assembly of daughter cell microtubules and the IMC, and before the mother cell’s cytoskeleton disassembles (Hu, 2008). DNA replication proceeds during S phase until the parasite is about 1.8N, which is reached right before daughter cells emerge (Radke et al., 2001; Blader et al., 2015). Karyokinesis is finally completed when the endoplasmic reticulum has finished dividing and the subpellicular microtubules which give the new parasites shape and structure have reached far into the growing daughter cell(s) (Radke et al., 2001; Blader et al., 2015). Above the subpellicular microtubules lies the inner membrane complex, a system of flattened rectangular membranous sacs that encircle the entire parasite, providing shape and structure, with openings at the apical and basal end (Bommer et al., 1968; Anderson-White et al., 2012).

Work in *Toxoplasma gondii* identified the basal complex as electron dense ring-shaped structures at the basal end of nascent parasites (Gubbels et al., 2006; Hu, 2008; Anderson-White et al., 2011; Hammarton, 2019). This basal complex, which accumulates at the leading edge of the developing daughter cell as it buds, is first marked by the unique localization of TgMORN1 (TGME49_310440), which occupies the basal gap of the inner membrane complex and constricts until it reaches the basal pole (Hu, 2008; Anderson-White et al., 2012). The formation and integrity of the inner membrane complex, or IMC, is thought to be connected to the function of the basal complex as it is essential for the proper formation of parasites during endodyogeny (Anderson-White et al., 2012; Dubey et al., 2017). Though its actual function is unknown, TgMORN1 is the earliest known marker of the *T. gondii* basal complex, and likely one of the first cytoskeletal structures assembled. TgMORN1 forms rings around duplicated centrioles before polarization occurs, significantly contributes to the structure of the complex, and co-localizes with MyoC, a noncanonical myosin which may, in conjunction with TgMORN1, provide the force necessary for basal complex constriction (**Figure 1**) (Gubbels et al., 2006; Hu, 2008). TgMORN1 is not unique to the basal complex, however; it also localizes to the centrocone, a structure that helps to organize the mitotic spindle in apicomplexans (Gubbels et al., 2006; Naumov et al., 2017).

In *T. gondii*, MORN1 and other basal complex proteins likely remain at the growing end of the cortical cytoskeleton, driven toward the basal end of the parasite by the addition of tubulin monomers to the subpellicular microtubules (Gubbels et al., 2006). However, TgMORN1 rings can assemble without cortical cytoskeleton growth, suggesting their maintenance is independent of, yet simultaneous with, cortical cytoskeleton assembly (Gubbels et al., 2006; Hu, 2008).

A host of other proteins have been identified as members of the *T. gondii* basal complex: TgHAD2a, TgDLC, TgCentrin2, TgIMC5, TgIMC8, TgIMC9, TgIMC13, TgIMC15, Tg14-3-3, TgMSC1a, TgMyoC, and TgMyoJ (Delbac et al., 2001; Hu, 2008; Lorestani et al., 2010; Anderson-White et al., 2011; Engelberg et al., 2016). However, only a few have been shown to be essential and/or are likely to play a role in generating contractile force.

TgCentrin2 (TGME49_250340*)* is recruited to the basal complex at the end of interphase, and occupies a compartment posterior (i.e. more basal) to that containing TgMORN1 (**Figure 1**). TgCentrin2 is hypothesized to contribute to contraction and may respond to changes in local Ca2+ concentrations *via* its EF-hand domain (Hu, 2008). Since elevating intracellular Ca2+ levels can artificially induce basal complex constriction, TgCentrin2 could drive this process.

TgDLC (TGME49_223000), a dynein light chain protein, and TgMyoJ (TGME49_257470), a non-canonical myosin, also localize to the basal complex (Hu, 2008; Frénal et al., 2017). TgMyoJ colocalizes with TgCentrin2 and in its absence, TgCentrin2 localization is abrogated, and constriction fails. Basal complex constriction also fails when TgCentrin2 is knocked down, suggesting both proteins are required for the basal complex to function appropriately (Frénal et al., 2017).

Destabilization of actin networks with cytochalasin D in *T. gondii* prevented both constriction and TgCentrin2 localization, suggesting basal complex formation and function depends on actin dynamics (Frénal et al., 2017). Though the specific roles of both actin and centrin2 in Toxoplasma are still unclear, recent research has broadened our understanding of their role in division. In the case of actin, since the actin cytoskeleton is composed of fibers that are inherently shorter and less stable than those of other organisms, it has been difficult to visualize actin *in vivo* in Apicomplexans. Recent work utilizing an actin chromobody has highlighted that while actin may be involved in the process of division, it seems to not form an actin ring at the basal end of the parasite (Periz et al., 2017). Basal morphology is negatively impacted by the loss of actin, but actin localizes to first the IMC and then the residual body over the course of Toxoplasma division; therefore the function of actin in the process of contractile ring formation and function is likely distinct from its key structural role in other eukaryotic contractile ring systems (Periz et al., 2017). In the case of Centrin2, the protein localizes to multiple locations within the parasite, including the preconoidal ring at the apical end, the basal complex, the centrioles, and peripheral annuli (Arnot et al., 2011; Leung et al., 2019). While division defects that may reflect a loss of basal complex activity are present in Cen2 knockdown parasites, it is difficult to assign these defects to the basal complex specifically because of the complex localization of Cen2 (Arnot et al., 2011; Leung et al., 2019). Within the basal complex, TgCentrin2 is hypothesized to act as a light chain for TgMyoJ, but this has not yet been demonstrated (Hu, 2008; Frénal et al., 2017).

### The *Plasmodium falciparum* Basal Complex

The basal complex and its composite proteins are less well characterized in *Plasmodium* than in *Toxoplasma*, and while both are Apicomplexans, during the asexual stage, *Toxoplasma* divides *via* endodyogeny, whereas *Plasmodium* asexual replication utilizes schizogony (as does *Toxoplasma* during the sexual stage). During schizogony, parasite nuclei divide asynchronously within the mother parasite, generating a multi-nucleated cell. The inner membrane complex, which in *Plasmodium* consists of a coherent vesicle rather than a series of plates, still provides shape and structure to the merozoites produced by schizogony, and the basal complex do not form until the final semi-synchronous round of nuclear division (**Figure 2**) (Harding and Meissner, 2014; Rudlaff et al., 2020). In this final division, the basal complex is thought to draw the inner membrane complex down around each merozoite, generating 18-32 new parasites. Both Apicomplexans, however, possess putative contractile rings that draw the inner membrane complex around the parasite and share some basal complex proteins.

*Plasmodium* MORN1 (PfMORN1; PF3D7_1031200), a homologue of TgMORN1, localizes to the basal complex only while TgMORN1 also localizes to the apical complex, a structure unique to and defining of the Apicomplexa which is located at the apical end of the cell and is involved in the invasion of host cells (Dubremetz et al., 1998; Ferguson et al., 2008; Katris et al., 2014) (**Figure 2**). PfCentrin2 (PF3D7_1446600) and PfMyoJ (PF3D7_1229800), whose homologs could be generating constrictive force in *Toxoplasma*, have been identified in co-immunoprecipitation experiments with basal complex proteins (Rudlaff et al., 2019). However, while TgCentrin2 localizes clearly to the basal complex, early studies demonstrate that PfCentrin2 localizes to the centrosome and is more strongly associated with the nucleus (Mahajan et al., 2008). Considering its interaction with basal complex proteins, *Plasmodium* Centrin 2 either indirectly assists in basal complex formation and function or further analysis or higher resolution imaging may be needed to determine the exact localization of PfCentrin2 during schizogony.

Further complicating Apicomplexan contractile ring theories is that knocking out the MyoJ ortholog PBANKA_1444500 in *P. berghei* does not inhibit basal complex constriction phenotype nor successful schizogony (Wall et al., 2019). Though transcriptional data suggests it to be highly expressed in schizogony, PbMyoJ-GFP protein was undetectable *via* immunofluorescence. We note that the mass spectrometry identification of PfMyoJ as an interacting partner of basal complex protein PfCINCH during schizogony demonstrates that in *P. falciparum*, MyoJ is expressed here (Rudlaff et al., 2019). In contrast to PbMyoJ, the *P. berghei* orthologs of MyoA, MyoF, and MyoK were all essential to asexual division, although neither are likely basal complex myosin candidates  (Wall et al., 2019). The function of PfMyoA (PF3D7_1342600) as part of the motor for parasite invasion, for example, has been well-established (Baum et al., 2006; Jones et al., 2006; Bookwalter et al., 2017; Perrin et al., 2018; Robert-Paganin et al., 2019). Therefore, whether the centrin and/or myosin-based force generation hypotheses for the *Toxoplasma* basal complex apply to *Plasmodium* remains unknown.

Other identified *Plasmodium* basal complex proteins are specific to the genus, such as PfBCP1 (PF3D7_1436200), PfBTP1 (PF3D7_0611600) and 2 (PF3D7_0704100), PfHAD2 (PF3D7_1205200) and PfCINCH (PF3D7_0407800), which interacts or at least colocalizes with these proteins, as well as PfMORN1 (**Figure 2**) (Guggisberg et al., 2017). We note that there is another *Plasmodium* protein that has also been named HAD2 (PF3D7_1226300) that is unrelated to the basal complex (Engelberg et al., 2016). PfCINCH is essential to *Plasmodium* cell division and is a crucial member of the basal complex, although its molecular mechanism remains unknown (Rudlaff et al., 2019). The importance of a *Plasmodium* specific protein potentially suggests that basal complex assembly and constriction mechanisms differ among Apicomplexa.

As in *Toxoplasma*, the structure and complete protein composition of the basal complex have not yet been uncovered, but actin dynamics may play a role. The best characterized role for actomyosin in *Plasmodium* is in gliding motility and invasion, which uses the motor activity of MyoA (Baum et al., 2006; Robert-Paganin et al., 2019). However, there is evidence for actin and associated proteins being involved in division also. PfACT1 (PF3D7_1246200) and multiple actin binding proteins present in *Plasmodium* – profilin, cofilin, CAP, ADF1, ADF2, and others – are upregulated in schizogony (Gordon and Sibley, 2005; Wong et al., 2011). Actin filaments are already known to play a role in the segregation of apicoplasts, vestigial plant-like plastids that are essential for apicomplexan growth and replication (Lim and McFadden, 2010). PfACT1 and PfFRM2 (PF3D7_1219000) (actin I and formin homologs respectively) knockdown parasites fail to segment or segregate apicoplasts (Das et al., 2017). Without actin I, merozoites are malformed and conjoined, lacking individual inner membrane complexes, indicating a role for actin in separating nascent merozoites, and thus likely in basal complex formation and function (Ganter et al., 2015; Das et al., 2017). Formin 2’s nucleating activity in *Plasmodium* stabilizes the inherently unstable actin polymers created by the divergent PfActin, and its upregulation during and requirement for schizogony suggests actin and actin stabilizing proteins may play a role in the formation and function of the basal complex (Stortz et al., 2019).

## The Most Well Characterized Contractile Rings: *S. pombe* and *S. cerevisiae* Models

The most extensive studies of the role of the cytokinetic contractile ring have occurred in yeast, specifically *S. pombe* and *S. cerevisiae*, making them an important comparand for studying the basal complex, and trying to determine how and *via* what mechanisms these rings function. In *S. pombe*, a species of yeast that divides by binary fission, contractile ring assembly and contraction have been studied in exquisite detail. First, the location for the ring is chosen by anillin-like Mid1p/Dmf1p which binds to the membrane in a series of nodes (Hachet and Simanis, 2008). This band of nodes is joined by myosin II heavy chain Myo2p, light chains Cdc4 and Rlc1, IQGAP, Rng2p, F-BAR protein Cdc15p, and formin Cdc12p (Laporte et al., 2010) (**Table 1**).

Though it divides by budding instead of fission, *S. cerevisiae* ring constriction/construction utilizes many *S. pombe* orthologs (Meitinger and Palani, 2016). Septins first assemble at the site of the bud neck, recruited by Rho-GTPase Cdc42, and form hetero-octameric filaments in a collar-like structure, which will surround the actomyosin ring (Wu et al., 2010; Oh and Bi, 2011). This scaffold recruits Bni5 which crosslinks the septin filaments and links with myosin light chain I, which in turn recruits myosin II with IQGAP homolog Iqg1 (Patasi et al., 2015) (**Table 1**). The importance of septins to contractile ring formation is species specific, however: in *S. cerevisiae*, Myo1p, the only type II myosin in this species, is recruited early on in formation of the contractile ring, recruited by septins (Bi et al., 1998; Lippincott and Li, 1998), whereas in *S. pombe*, Myo2 is recruited well before septins, which are nonessential for viability (Wu et al., 2003).

Myosin motors are involved in contractile ring assembly in both *S. pombe* and *S. cerevisiae*, but there are important differences. *S. cerevisiae*’s Myo1p appears at the ring quite early and before actin. In *S. pombe*, two class II and one class V myosin are required for assembly and contraction. Myo2, the conventional myosin-II homolog which is primarily responsible for assembly and contraction, is recruited first, before actin polymerization is initiated by Cdc12. Myo51, the class V myosin, is recruited once actin polymerization and node condensing begin, and Myp2 is recruited only once the contractile rings are fully formed, though each can form nodes and assemble the ring in the absence of the others (Laplante et al., 2015).

**Figure 3:**
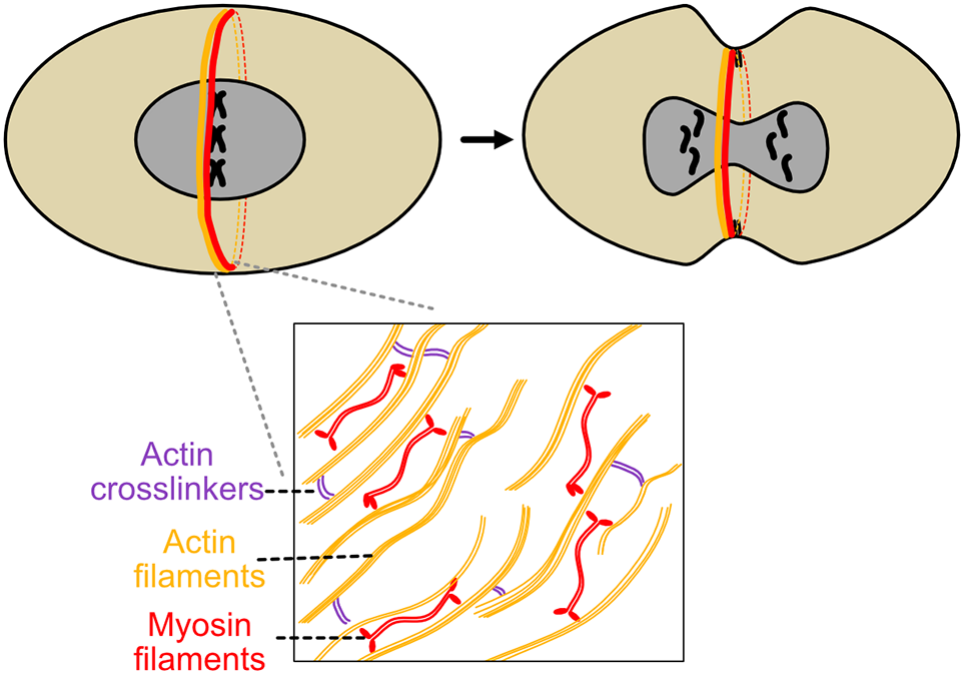
Actomyosin ring constriction during *S. pombe* cytokinesis. *S. pombe*’s contractile ring is perhaps the most extensively studied example of this structure. Actin and myosin are recruited to the nascent ring, actin filament directionality is modified by formins, and myosin II ATPase activity in conjunction with actin dynamics generates constrictive force. As constriction proceeds, glucan synthesis by bgs1 helps induce septum ingression and gives the nascent cell shape and structure.

Initially, there are two distinct groups of parallel actin filaments of opposite directionality, arrayed in a semicircle (Kamasaki et al., 2007). After assembly, actin directionality becomes mixed, expedited by formin Cdc12p nucleation of actin filaments (Coffman et al., 2013). During contraction, populations of f-actin slide over each other, then depolymerize during contraction, which itself generates force (Kamasaki et al., 2007) (**Figure 3**). This action is capable of enabling ring constriction in *S. cerevisae* on its own, but in *S. pombe*, myosin 2 ATPase activity is required for the process of cytokinesis (Kitayama et al., 1997, Lord et al., 2005).

Apicomplexans lack myosin II and tropomyosin. While their roles in *Plasmodium* are still unclear, in *Toxoplasma*, both MyoJ and Centrin 2 are involved in constricting the basal complex, and it is hypothesized that the EF-hand domain in TgCen2 could allow it to act as a myosin light chain for TgMyoJ and generate contractile force (Geimer and Melkonian, 2005; Frénal et al., 2017). In *S. pombe*, two unconventional myosins are utilized during division, Myp2p and Myo51. In particular, the fact the yeast can complete cytokinesis with Myo51 only does speak to the possibility of non-myosin II myosins being capable of providing force for cytokinetic ring constriction (Laplante et al., 2015).

Actin polymerization in *S. pombe*, required for contractile ring assembly and function, requires Arp2/3, Cdc12/For3, profilin, cofilin, and WASP – but not necessarily myosin II or IQGAP, as rings, albeit defective, can form even without myosin II nodes (Bathe and Chang, 2010). In *S. cerevisiae*, actin *de*polymerization may be the predominant mechanism underlying contraction (Mendes Pinto et al., 2012). Considering actin polymerization/depolymerization as a force generating mechanism; *Plasmodium does* have two formin proteins; one has homology to Cdc12 and the other to For3 (Baum et al., 2008). The former functions during invasion but the latter is highly expressed in late schizogony and could act in *Plasmodium*’s basal complex like For3 in the *S. pombe* contractile ring. *Plasmodium* also contains a cofilin homolog (PF3D7_1361400), capable of binding actin monomers and upregulated in late schizogony (**Table 1**). However, proteins which stabilize actin filaments (tropomyosin, a-actinin) lack apicomplexan homologs, so if the short, transient actin filaments of apicomplexans form the structure of the contractile ring, novel proteins are likely required for assembly and constriction.

Constriction speeds as anaphase concludes, and cytokinesis begins to occur in conjunction with this transition (Willet et al., 2015). Glucan (*S. pombe*) or chitin (*S. cerevisiae*) synthesizing proteins are recruited to the ring (Santos et al., 2003; Hercyk et al., 2019). These proteins induce ingression of the septum and membrane invagination, processes which are not only coupled to ring constriction, but also required for proper placement of the ring (Willet et al., 2015).

In yeast, ring constriction drives septum formation. In Apicomplexan division, while it is not known if the IMC is built up simultaneously with basal complex constriction, the basal complex assembles independently of microtubules and the IMC (Hu, 2008). The basal complex and apical complex are, however, separated as microtubules polymerize and the daughter cells grow, and in *Toxoplasma*, the constriction of the basal complex brings IMC plates together. During the asexual blood stage, however, the IMC in *Plasmodium* lacks plates and is made of a single vesicle (Bannister and Mitchell, 1995). It remains unknown if the ring drives formation of furrows between *Plasmodium* merozoites, for example, or the expansion of the IMC, as the cytokinetic ring drives formation of the furrow and septum ingression in *S. pombe.*

## Contractile Rings in Metazoan Cytokinesis

While yeast cytokinesis may be the most well studied, the mechanisms of cytokinesis and the processes of contractile ring assembly can be more complicated in metazoans, if not in basic replicative cytokinesis, then in the creation of multiple cells from one predecessor during embryogenesis (Pollard, 2017). Thus, comparing Apicomplexan contractile rings to their metazoan counterparts may reveal similarities on this basis, since Apicomplexans also produce multiple progeny during both asexual and sexual division.

As in yeast, animal cell contractile rings consist of actin filaments, bipolar myosin II filaments, and septin filaments. Anillin crosslinkers can bind all three filament types, and other proteins which modulate actin dynamics play a similarly important role (D’Avino, 2009). Contractile ring assembly is controlled temporally by mitotic kinase levels. During metaphase, MKLP-1 is phosphorylated by Cdk1/Cyclin b, inhibiting its ability to bind microtubules (Kamijo et al., 2005). When anaphase begins, Cdk1 activity declines, phosphorylation cannot be maintained, and centralspindlin binds microtubules, in a process mediated by Aurora B kinase and 14-3-3 proteins (Basant et al., 2015). Centralspindlin interacts with Ect2, which enhances Rho turnover, allowing it to quickly cycle between inactive (GDP-bound) and active (GTP-bound) forms (Mishima et al, 2002). Active, GTP-bound Rho interacts with formins to polymerize actin and with myosin light chains to induce myosin II activation/filament formation at the equatorial cortex, generating the actin and myosin II filaments which form the contractile ring (**Table 1**) (Maupin and Pollard, 1986; Piekny et al., 2005; Makkonen et al., 2013).

Many of these kinases and phosphorylases involved in contractile ring assembly lack Apicomplexan orthologs, but both *Toxoplasma* and *Plasmodium* contain three Aurora-related Kinases (TgArk 1-3: TGME49_210280, TGME49_318770, TGME49_203010; PfArk 1-3: PF3D7_0605300, PF3D7_0309200, PF3D7_1356800) (**Table 1**) (Berry et al., 2016). While Aurora B kinase in animal cells helps recruit proteins to the nascent contractile ring none of the *Plasmodium* homologs localize to the basal complex, seemingly associated with other novel cytoskeleton development during cytokinesis (Berry et al., 2016).

Neither of the *Toxoplasma* Aurora kinase orthologs localize with TgMORN1 either, but if cytoskeletal assembly coordinated by the centrioles is involved in basal complex formation, it could be that Pf/TgArk plays an indirect role in basal complex formation. In the absence of TgArk3, *T. gondii* parasites failed to form the typical “rosette” structure of dividing tachyzoites, a morphological defect that could indicate a role for Apicomplexan aurora kinases in organizing the basal complex (Berry et al., 2016). Basal complex integrity needs to be examined in Ark-knockdown parasites to determine if Pf/TgArks are involved in its assembly and function.

Once all proteins have been recruited to the nascent metazoan actomyosin ring, assembly ends, and cytokinesis begins. Actin modulating proteins like profilin, formins, cofilin, and anillin parallel their yeast counterparts during ring constriction (Hachet and Simanis, 2008; Piekny and Glotzer, 2008). The ring contracts when bipolar myosin II filaments slide along antiparallel actin filaments. F-actin disassembly balances polymerization; both are required for contraction (Khaliullin et al., 2018; Pollard and O’Shaughnessy, 2019). Though metazoan contractile ring force generation mechanisms have not been fully elucidated, it is posited that either myosin II generates contractile stress around bundles of randomly-oriented actin filaments, or that contractile stress is generated by actin dynamics alone (Oelz et al., 2015; Pollard and O’Shaughnessy, 2019).

As mentioned, some metazoan systems utilize more complex cell division processes. For example, during *Drosophila melanogaster* embryonic development, the multi-nucleated syncytial blastoderm divides to form several individual cells, each with a single nucleus, in a process known as cellularization (Mazumdar and Mazumdar, 2002). Like Apicomplexan schizogony, cellularization relies on a contractile ring to draw down membranes and create individual cells from a collection of nuclei (Chougule et al., 2016; Carter et al., 2019). There are two “phases” of ring constriction during cellularization, whose contributions depend on initial ring size (Martin, 2016; Xue and Sokac, 2016). Since initial ring size determines constriction rate, the contractile ring must retain a ‘structural memory’ of its initial size, allowing it to constrict as fast as required for successful mitosis (Carvalho et al., 2009). Thus, it is thought that the ring is assembled from a number of ‘contractile units’ of fixed size and which shorten at a constant rate according to actin depolymerization (Carvalho et al., 2009). This could be compared to Apicomplexan schizogony – while each merozoite tends to be the same size, the final number of merozoites formed depends on how many merozoites the parasite ‘chose’ to make before its final division. Rings formed of contractile units could assemble in as great a number as necessary to surround each merozoite, and still constrict contemporaneously.

## Contractile Action in Mitochondrial Division

Considering the divergent nature of apicomplexan biology, it isn’t surprising that there are striking similarities between apicomplexan contractile rings/division structures and those required in more ancient forms of division. Whether or not a contractile ring is involved in the process of mitochondrial fission, many of the proteins utilized in actomyosin ring – mediated cytokinesis in eukaryotic cells are also involved in mitochondrial fission, from myosin II to septins to actin modulating proteins such as Arp2/3, cofilin, and cortactin (**Table 1**) (Mears et al., 2011; Pagliuso et al., 2018).

The primary and best-characterized action of mitochondrial fission relies on the action of dynamin related protein Drp1 which regulates constriction and remodeling of the mitochondria *via* oligomerization (Mears et al., 2011). Drp1 is a GTPase; upon the hydrolysis of GTP, a conformational change induces compaction of Drp1 multimeric spirals and constriction of the mitochondrion, supporting the idea of a contractile mechanism in fission (Mears et al., 2011). Further effectors of Drp1 recruitment are ER tubules which help determine the site of fission and the presence of actin and myosin (Mears et al., 2011).

Like in cytokinesis, septins regulate fission by interacting with Drp1 and as in cellularization, transient actin polymerization coincides with mitochondrial fission (Gandre-Babbe and van der Bliek, 2008; Pagliuso et al., 2016).

Mitochondrial fission relies on the constricting force of an actin and myosin II complex- as well as spiraling Drp1 oligomers regulated by septins, although dynamin performs the terminal abscission step (Pagliuso et al., 2016; Pagliuso et al., 2018).

This could be an attractive comparand for apicomplexan division: There are two functional dynamin proteins in *P. falciparum*, both upregulated during schizogony and homologous to human dynamin proteins (**Table 1**). PfDYN2 (PF3D7_1037500) contains the GTPase and GED domains consistent with dynamins but lacks the C-terminal PRD and domains associated with “classical” dynamins, resembling human Drp1 in structure (Charneau et al., 2007). PfDYN1 (PF3D7_1145400) has a longer C-terminal domain and localizes to membrane compartments whereas PfDYN2 forms punctate structures in the cytoplasm and colocalizes with the ER/Golgi body and apicoplast (Li et al., 2004; Charneau et al., 2007). While the necessity of PfDYN2 has not been determined, knockdown of PfDYN1 hindered parasite replication, thus the dynamins are not redundant (Li et al., 2004). Examining *Plasmodium* dynamins in late schizogony could help determine whether dynamin/DLPs are involved in the basal complex

In *Toxoplasma*, more is known about how dynamin-like proteins, particularly a Drp1 homolog called DrpC, contribute to overall division (**Table 1**) (Breinich et al., 2009; Heredero-Bermejo et al., 2019). Like *Plasmodium* DYN2, TgDrpC (TGME49_270690) localizes to cytoplasmic puncta, but in dividing parasites it redistributes to the growing edge of daughter cells. TgDrpC also co-localizes with TgMORN1, an early marker of the *Toxoplasma* basal complex, and forms similar ring structures. Conditional knockdown of TgDrpC induced mitochondrial fragmentation, but also caused defects in apicoplast division and IMC formation which altogether halted division, suggesting a fundamental role for DrpC in endodyogeny (Heredero-Bermejo et al., 2019). Like TgMORN1, TgDrpC normally relocates from the cytoplasm to ring-like structures at the growing ends of daughter buds, but the phenotypic consequences of the TgDrpC knockdown are quite broad, so while this could suggest a role within the basal complex for DrpC, it may also be the result of broader defects in endodyogeny than a direct relationship with the basal complex.

However, there are complicating factors, namely a separate paper wherein conditional knockdown of DrpC did not impact any organelles other than the mitochondria (Melatti et al., 2019). While it is possible that TgDrpC could, like Drp1 during mitochondrial fission, recruit proteins to help form the contractile ring and generate force, conflicting information that may result from different strategies used to generate DrpC knockdown lines or the genetic modifications (ie, addition of fluorescent proteins) themselves makes it difficult to make a true prediction without more information (Heredero-Bermejo et al., 2019; Melatti et al., 2019). TgDrpC is conserved in *Plasmodium* as protein PF3D7_1218500, which has a similar transcriptomic expression to basal complex proteins. Further studies on this *Plasmodium* homolog (PfDrpC) will determine if its functions in *Plasmodium* mirror those in *Toxoplasma*.

In *Toxoplasma* as well, some proteins in the basal complex are also directly involved in the division of the apicoplast, but making direct conclusions is difficult considering the lack of mechanistic knowledge as yet. TgMORN1 is required for successful apicoplast segregation, and it had been hypothesized that the growing daughter cell, led at the basal end by the constricting TgMORN1-containing basal complex, has a functional role in apicoplast division and generates the force for apicoplast fission (van Dooren et al., 2009). However, while MORN1 is essential for apicoplast segregation, DrpA is likely primarily responsible for generating force, as DrpA’s knockdown did not inhibit general cell division, but did inhibit apicoplast fission (van Dooren et al., 2009). TgMORN1 does not surround the apicoplast tightly and any specific role in recruiting DrpA or constricting the apicoplast has not been determined; though it is clearly relevant to the process, it may not be directly related to its role in the basal complex, and it is likely dynamin-like protein DrpA which performs the final separation (van Dooren et al., 2009; Lorestani et al., 2010). While TgMORN1 is required, its specific role in apicoplast division, whether or not it is tied to its basal complex functions, remains to be determined.

With these caveats outlined, it is true that in TgMORN1-deficient parasites, not only did apicoplast segregation fail, but the tachyzoite daughters themselves failed to separate/constrict  (Heaslip et al., 2010; Lorestani et al., 2010). The fact that, in *Toxoplasma*, the apicoplast and the entire parasite utilize at least one significant, shared protein between them (TgMORN1) to divide, and both division processes require dynamin-related proteins, suggests that the basal complex may function similarly to contractile ring structures in mitochondrial division, even if the proteins utilized in each process vary. However, this hypothesis illustrates the necessity of considering Apicomplexan diversity: in *Plasmodium*, PfMORN1localizes only to the basal complex; thus apicoplast segregation may not be connected at all to the constriction of the basal complex

## The Contractile Ring in *Florideophyte* Red Algae

Though there may be interesting points of comparison between Apicomplexans and Opisthokontae (a clade containing both the fungi and animal kingdoms), Apicomplexans are extremely phylogenetically distant from them. Looking outside of Opisthokonta for well-studied organisms, we could compare Apicomplexan contractile rings to those of red algae, which are much more closely related to the Alveolata, which includes Apicomplexans. Indeed, red algae and alveolates are contained within the same recently- described “megagroup”, distinct from that containing fungi and animals (Burki et al., 2008).

Contractile rings for division exist in florideophyte red algae, whose division may resemble the separation of *Plasmodium* merozoites after multiple karyokineses (Garbary and McDonald, 1996). Florideophyte actin rings are formed early in cytokinesis, and in the *Tiffaniella/Griffithsia* genera, nuclei move out of the furrowing zone in preparation for the formation of the actin ring, although specific mechanisms remain unknown (Garbary and McDonald, 1996).

In *C. merolae*, the contractile ring used to divide the chloroplast has been studied with a significant degree of detail, and much more is known about its formation, function, and accessory proteins (Kuroiwa, 1998; Sumiya et al., 2014; Yoshida and Mogi, 2019). Plastid dividing rings contain a dynamic ‘trio’ of rings: the PD or MD ring (PDR1/MDR1), the FtsZ ring (FtsZ1/2 or A/B), and the dynamin ring (Dnm2), which spans the double membrane (Osteryoung and Pyke, 2014; Yoshida, 2018). While few of these proteins or their have direct Apicomplexan orthologs, microtubule-like FtsZ and dynamin-like DNM2 may provide intriguing comparands (Imoto et al., 2011; TerBush et al., 2013) (**Table 1**).

In bacteria, FtsZ forms rings at the cell midpoint. In chloroplasts, eukaryotic homologs FtsZA and B work together to form the FtsZ ring. They form protofilaments which bundle into the ring in a 1:1 ratio, similar to microtubule assembly (Yoshida et al., 2016). This outer ring in conjunction with PDR1 then recruits Dnm2, a dynamin-related protein, and helps cross-link outer PD ring filaments (Miyagishima et al., 2008). While Dnm2 is essential for plastid division, it, like *Plasmodium* Dyn1/Dyn2 and *Toxoplasma* DrpC, only has GTP binding/hydrolyzing domains, unlike conventional dynamins (**Table 1**). Apicomplexan dynamins could therefore be capable of playing a similar role in ring formation and contraction during schizogony (Miyagishima et al., 2008).

Mechanisms for contraction of this triple-ring system are unknown, but it is hypothesized that FtsZ and Dnm2 generate force and Dnm2’s GTP hydrolysis induces destabilization, disassembly, and release of FtsZ monomers (Yoshida et al., 2016; Yoshida, 2018). These actin and myosin- free contraction models may merit further study in the Apicomplexan context as well - Dnm2/DRP5B aligns with *Plasmodium* DYN1, and Dnm1 with *Plasmodium* DYN2, strengthening the idea that these proteins could generate constrictive force in the basal complex in the absence of myosin II (**Table 1**).

While many *C. merolae* plastid division proteins lack Apicomplexan orthologs, Arc6, a protein required for FtsZ ring assembly, has multiple orthologs in *Babesia*, another Apicomplexan. A search of this protein sequence in the *Plasmodium* database reveals PF3D7_0803200, a putative filament-former with exactly analogous timing to known basal complex proteins (**Table 1**). As the protein is completely uncharacterized, it would be interesting to determine if it functions similarly in the assembly of the basal complex, as its timing and homology suggests.

## Cytokinetic Contractile Rings and Other Contractile Mechanisms in *Dictyostelium*

The slime mold *Dictyostelium* may be another useful model for the role of a contractile ring in Apicomplexan division. Amoebozoa are more distantly related to Apicomplexa than red algae, but *Dictyostelium* contains well-studied examples of myosin II-free cytokinetic division. *Dictyostelium* can perform 4 types of cytokinesis but only cytokinesis A involves a contractile ring. Though myosin II is required for cytokinesis A, associated proteins do have Apicomplexan homologs (Yumura, 2001). *Dictyostelium* has five dynamin-like proteins: DymA and DymB have a GTPase domain and a GTPase effector domain, like *Plasmodium* DYN1/2. DlpA, B, and C, have a GTPase domain only, like *Toxoplasma* DrpC (**Table 1**). All three of the dynamins involved in cytokinesis in *Dictyostelium* have GTPase domains required for cytokinesis (Rai et al., 2011; Rana et al., 2013). The role of dynamin-like proteins in cytokinesis A depends on their GTPase activity, and in *Plasmodium* and *Toxoplasma* all dynamin homologs have the GTPase domain conserved as well. DlpB and A both contribute to furrowing; they colocalize and in their absence, actin filaments fragment (Rai et al., 2011; Fujimoto et al., 2019). Reliance on stabilized actin filaments for division may seem an unlikely model for Apicomplexans where short, unstable actin filaments proliferate, but *Plasmodium/Toxoplasma* DrpC could perform a similarly stabilizing role (Rana et al., 2013).

*Dictyostelium* wound closure may also compare to *Plasmodium* asexual schizogony or *Toxoplasma* sexual schizogony in particular: in other eukaryotes, such as the *D. melanogaster* embryo, actomyosin ring contraction at the leading edge of the wound closes it but *Dictyostelium* utilizes a myosin independent ‘contractile ring’ reliant on calcium signaling (Darenfed and Mandato, 2005; Abreu-Blanco et al., 2012). Actin accumulates at the wound site filaments and forms a ring like structure, then a filled circle that grows and then decreases (Itoh and Yumura, 2007). Actin accumulation is dependent on Ca2+ signaling preceding it, just as *Plasmodium* schizogony relies on calcium signaling (Talukder et al., 2020). In the presence of latrunculin A, membrane pore size increases – thus therefore, actin may form a constrictive ring around the pore. This model where a contractile ring of actin leads a sheet of new membrane to fill the pore may echo how the *Plasmodium* basal complex draws down the IMC during schizogony (Talukder et al., 2020).

## Discussion

Apicomplexan methods of cell division are profoundly distinct from those of other eukaryotes, making it difficult to elucidate their underlying mechanisms. The basal complex, present in *Toxoplasma* and *Plasmodium*, is integral to division but poorly understood as a result of Apicomplexan evolutionary divergence. By comparing what *is* known about the basal complex to well-studied contractile rings, it is hoped that possible comparands to the Apicomplexan basal complex could help guide research directions.

If the basal complex is a variation on an actomyosin contractile ring, one of the many non-canonical myosins in Apicomplexans will have to act as myosin II. In *Toxoplasma*, TgMyoJ is already hypothesized to help generate constrictive force. *Plasmodium* MyoJ, however, did not localize to the basal complex and is not predicted to be essential. *Plasmodium* MyoJ and Cen2’s relationship to other basal complex proteins should be re-examined, but there may be different methods of force generation among Apicomplexans.

Careful examination of contractile rings among eukaryotes can reveal interesting parallels such as between asexual Apicomplexan and mitochondrial division. Dynamin-like protein DrpC has already been shown to be required for successful *Toxoplasma* endodyogeny. Further study on the localization and function of its homolog in *Plasmodium*, and other dynamin like proteins identified and only partially characterized, could elucidate additional molecular details.

The biphasic constriction model identified in the process of embryo cellularization in *Drosophila melanogaster* can suggest mechanisms for the generation of constrictive force in the absence of canonical myosins in Apicomplexans. Apicomplexans contain multiple proteins capable of modulating actin dynamics, and a closer examination of the role of Actin I in cytokinesis could determine if actin depolymerization is largely responsible for constriction.

All of these are merely possibilities, hypothetical conclusions drawn from interesting similarities. We hope that they may serve as a rough starting point to understand basal complex function. In any case, it is clear that before the basal complex can be classified or even accurately compared with well-studied contractile rings in other systems, many more protein components of the complex must be identified. The newfound relative ease of genetic manipulation in both primary Apicomplexan genera, however, makes that task less daunting and more approachable than ever before.

## Author Contributions

AM and JD wrote the manuscript. All authors contributed to the article and approved the submitted version.

## Funding

The authors acknowledge funding from the National Institutes of Health to JD (R01 AI145941).

## Conflict of Interest

The authors declare that the research was conducted in the absence of any commercial or financial relationships that could be construed as a potential conflict of interest.
